# Pediatric heart failure in the era of cardio-immunology: inflammatory–immune context phenotypes, diagnosis, and targeted management

**DOI:** 10.3389/fimmu.2026.1776897

**Published:** 2026-04-20

**Authors:** Danna Li, Dezhen Yao

**Affiliations:** Pediatric Department of Shengjing Hospital of China Medical University, Shenyang, China

**Keywords:** adaptive immunity, biomarkers, cardio-immunology, cytokines, endothelial activation, inflammation, innate immunity, pediatric heart failure

## Abstract

Pediatric heart failure (PHF) is a heterogeneous syndrome whose etiologies, developmental biology, and clinical trajectories differ fundamentally from adult heart failure. Beyond age-specific hemodynamics and neurohormonal activation, inflammatory and immune programs can serve as primary drivers (e.g., myocarditis, multisystem inflammatory syndrome in children [MIS-C]) or as potent amplifiers of decompensation (e.g., postoperative inflammation after cardiopulmonary bypass, infection-associated stress). This review integrates contemporary pediatric heart failure practice with insights from cardio-immunology and proposes a pragmatic, bedside framework that: (i) links common triggers and clinical contexts to likely effector pathways; (ii) uses multimodal assessment to distinguish active immune-mediated injury from chronic remodeling; (iii) applies trajectory-based monitoring with feasible inflammatory–immune context bundles rather than research-grade immunophenotyping; and (iv) aligns immunomodulatory decisions with probability of immune causality, time window, and safety constraints to avoid indiscriminate immunosuppression. We highlight pediatric-specific challenges (reference ranges, sampling feasibility, imaging constraints, and limits of adult extrapolation), summarize emerging pharmacologic and device advances, and outline priorities for harmonized pediatric biomarker standards, prospective phenotype validation, and safer implementation of targeted immunomodulation. A graphical abstract summarizes the proposed phenotype-to-management framework.

## Graphical Abstract

**Figure:**
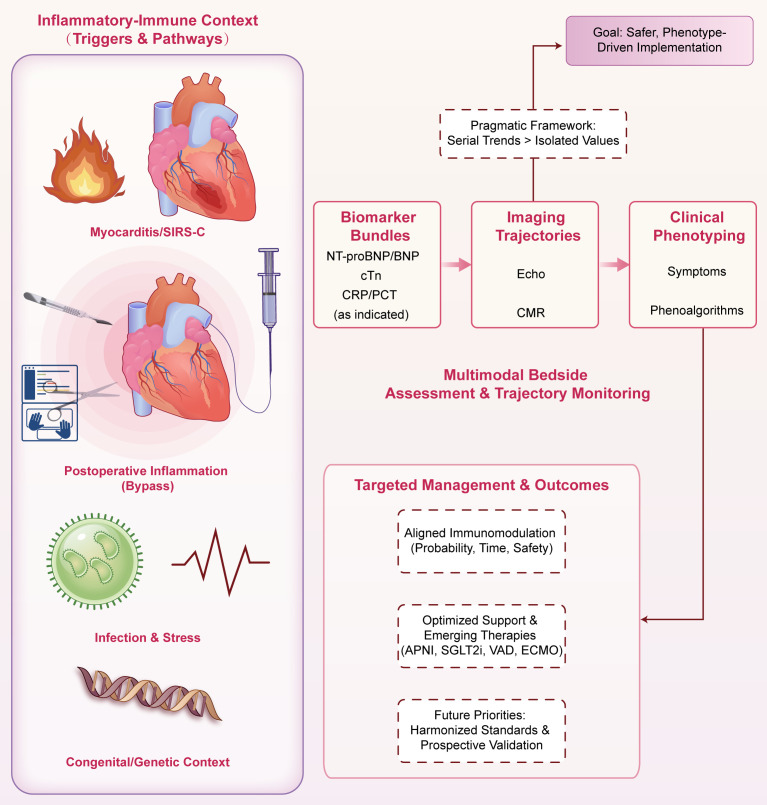

## 1 Introduction

### 1.1 What makes pediatric heart failure distinct

#### 1.1.1 Definition and clinical peculiarities of pediatric heart failure

Pediatric heart failure (PHF) is not simply a smaller-scale version of adult heart failure but a distinct clinical syndrome with unique etiologies, pathophysiological mechanisms, clinical manifestations, responses to treatment, and prognostic patterns (1, 2). It is generally defined as structural or functional cardiac abnormality leading to inadequate cardiac output to meet metabolic demands, or to maintain adequate output only at the expense of elevated filling pressures (3).

In contrast to adult heart failure, which is dominated by ischemic cardiomyopathy (4), pediatric heart failure is etiologically diverse and strongly age-dependent (5). In infancy, critical congenital heart disease (e.g., hypoplastic left heart syndrome, transposition of the great arteries) and genetic/metabolic cardiomyopathies predominate, including dilated, hypertrophic, and restrictive phenotypes and inborn errors of metabolism (e.g., fatty-acid oxidation defects) (6). Across childhood, acquired insults such as viral myocarditis, chemotherapy-related cardiotoxicity, and systemic inflammatory or autoimmune diseases contribute substantially (7, 8). Inherited arrhythmogenic cardiomyopathy is more often recognized in older children and adolescents, and neuromuscular disorders (e.g., Duchenne muscular dystrophy) remain an important secondary cardiomyopathy spectrum (9, 10).

The immature myocardium differs from the adult heart in contractile protein composition, calcium handling, receptor expression, and myocardial reserve (11–13). These developmental characteristics attenuate compensatory capacity under pressure or volume overload, enhance susceptibility to neurohormonal activation and inflammatory injury, and coexist with a partially preserved regenerative potential (14, 15). Such ontogeny-dependent features provide a strong rationale for pediatric-specific diagnostic and therapeutic algorithms rather than direct extrapolation from adult protocols (2, 15).

In addition to these structural and neurohormonal distinctions, PHF is embedded in an immuno-developmental context (16). The pediatric immune system is dynamically maturing (including thymic output, innate training, and evolving antibody repertoires), which can shape the balance between pathogen control, immune-mediated injury, and resolution (17, 18). This helps explain why inflammatory syndromes—such as myocarditis, MIS-C, and postoperative systemic inflammation—may precipitate abrupt decompensation or leave a disproportionate imprint on remodeling (19–21). A critical conceptual distinction throughout this review is the difference between the underlying etiology—the fixed or slowly evolving substrate that predisposes a child to heart failure (e.g., genetic cardiomyopathy, surgically corrected congenital heart disease)—and the immune context—the transient, reactive state that may superimpose upon that substrate and drive acute decompensation or accelerated progression (e.g., acute myocarditis, postoperative inflammation, infection-associated decompensation). These immune contexts are not fixed properties of a given etiology but rather dynamic states that can evolve over time, requiring serial reassessment and context-appropriate therapeutic responses. Accordingly, etiologic classification should be complemented by inflammatory–immune context assessment, linking clinical triggers and biomarkers to actionable windows for immunomodulation and intensified surveillance (22, 23).

Practical pediatric lens used throughout this review: (1) developmental stage shapes immune tone and myocardial reserve (24); (2) trigger and clinical context (e.g., myocarditis/MIS-C, postoperative inflammation, infection, congenital lesions) matters as much as ejection fraction (25); (3) feasibility and interpretation of tests require age-adjusted reference ranges and attention to timing/trajectory (26); and (4) treatment recommendations must reflect pediatric evidence boundaries and safety, rather than direct extrapolation from adult protocols (27).

### 1.2 Epidemiology, disease burden, and rationale for cardio-immunology

#### 1.2.1 Epidemiology

Although less common than adult heart failure, PHF carries substantial morbidity, mortality, and resource utilization (28, 29). The annual incidence has been estimated at approximately 0.87–7.4 per 100,000 children, with congenital heart disease (CHD) and cardiomyopathies accounting for the majority of cases (28, 30). Cardiomyopathy incidence is about 1.13 per 100,000 children and peaks in infancy (8.34 per 100,000), highlighting marked age-related vulnerability (10).

Population-based estimates suggest approximately 11,000–14,000 pediatric heart failure–related hospitalizations annually in the United States, with an overall in-hospital mortality around 7% (29). Mortality can exceed 20% in the presence of major comorbidities (e.g., renal failure), and infants experience disproportionately high risk (31). Cardiomyopathy and end-stage heart failure remain among the leading indications for pediatric heart transplantation across major registries, underscoring PHF as a low-incidence but high-impact condition in pediatric practice (32).

#### 1.2.2 Disease burden

The burden of PHF extends across patient, family, and healthcare system levels (33). At the patient level, chronic low cardiac output contributes to growth failure (weight/height Z-scores < −2) (34, 35), reduced exercise tolerance (peak VO_2_ reduction >40%) (36, 37), and neurocognitive impairment attributed to chronic cerebral hypoperfusion (38). Families often experience sustained psychosocial strain, and high rates of caregiver anxiety and depressive symptoms have been reported (39). Health-system impact is also substantial: PHF is associated with recurrent hospitalizations and prolonged intensive care use, particularly when advanced therapies such as ECMO, ventricular assist devices, or transplantation are required (40, 41).

#### 1.2.3 Rapidly evolving frontiers

In recent years, three major developments have reshaped the management of PHF. First, targeted genomic panel sequencing has significantly increased the diagnostic yield for cardiomyopathies and inherited cardiovascular disorders, enabling more precise etiologic classification and family counseling (42–44). Second, CMR T1 mapping and other advanced tissue characterization techniques allow noninvasive quantification of myocardial fibrosis and infiltration, refining prognostication and guiding therapy (45). Third, contemporary mechanical circulatory support devices have expanded therapeutic options (46). For example, a single-center series of pediatric patients (7–18 years) with end-stage heart failure supported by the HeartMate 3 (HM3) reported survival on device in 81.8% (9/11) and successful transplantation in 9.1% (1/11) over a median follow-up of 150 days (47). Pump thrombosis, ischemic stroke, and device malfunction were not observed; complications included pleural effusion, tamponade, and polyuria, and anticoagulation non-adherence resulted in right atrial thrombosis in one patient, which resolved after regimen optimization.

Pharmacologic advances are also emerging. In the PANORAMA-HF trial, sacubitril/valsartan did not significantly outperform enalapril on the primary clinical endpoint in pediatric patients with systemic LV systolic dysfunction, although both arms improved symptoms and NT-proBNP with comparable safety (48). In parallel, machine-learning models integrating genotypes (e.g., LMNA variants), circulating biomarkers (e.g., galectin-3, sST2), and CMR parameters are improving risk stratification for mortality and transplantation in heart failure populations (49).

**Figure 1:**
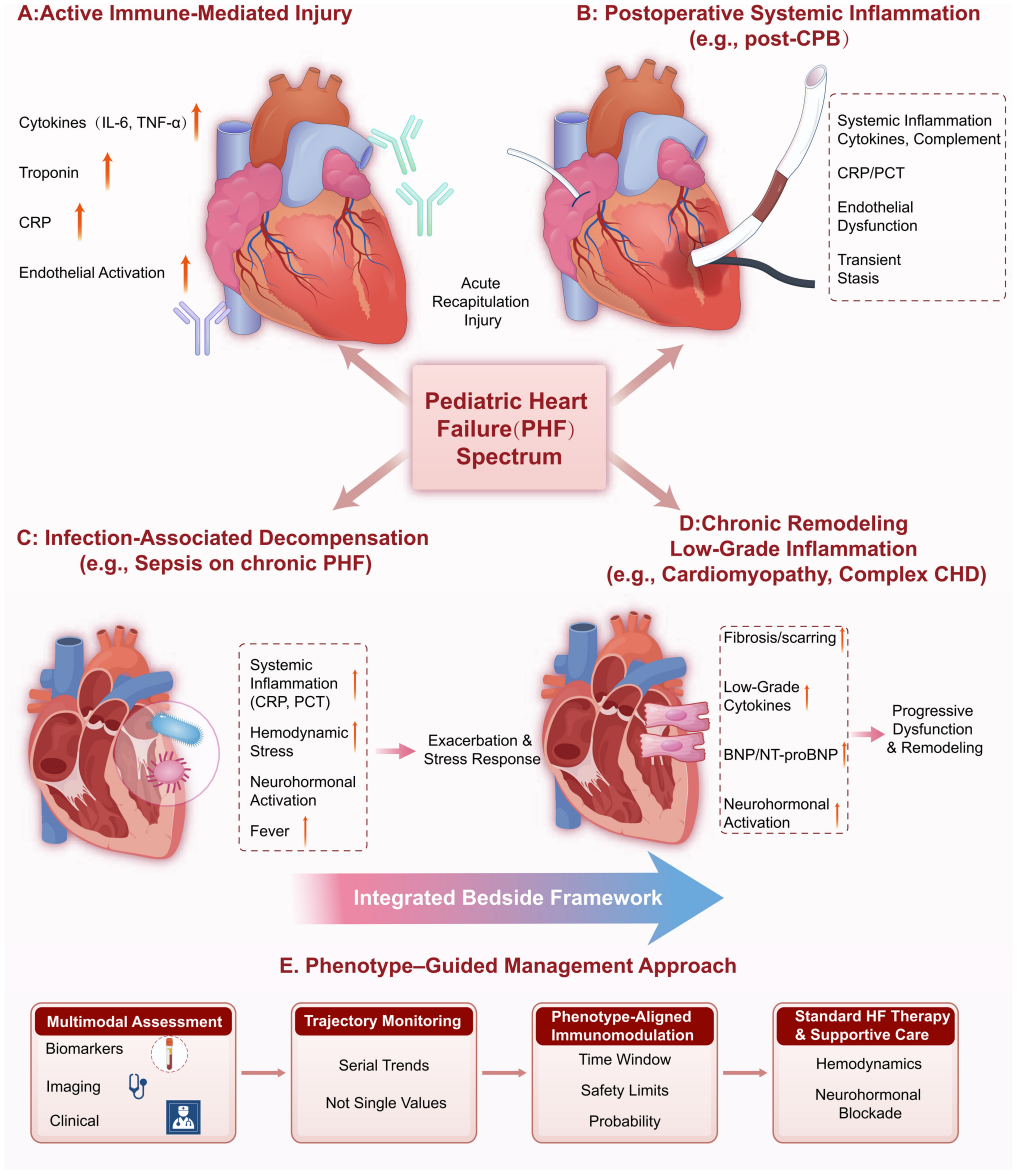
Inflammatory–immune context phenotypes in pediatric heart failure.

This review synthesizes pediatric-focused evidence and selected mechanistic adult literature to propose a pragmatic bedside framework for inflammatory–immune context phenotyping and management in pediatric heart failure. The proposed phenotype framework is summarized in **Figure 1**.

## 2 Pathophysiology: hemodynamics-to-immunity integration

The onset of heart failure triggers complex interactions among neurohormonal systems, inflammatory mediators, and tissue remodeling pathways that initially compensate for reduced cardiac output but ultimately contribute to disease progression (50). Compared with adults, children show distinct patterns of adrenergic receptor expression, microRNA signatures, and transcriptomic profiles, particularly in the setting of congenital structural heart disease and genetically determined cardiomyopathies (51). Immune activation is particularly relevant in infection-, surgery-, and myocarditis-related phenotypes, where cytokine signaling and endothelial dysfunction can accelerate decompensation and remodeling (52).

### 2.1 Neurohormonal activation

The sympathetic nervous system, through norepinephrine and epinephrine release, increases heart rate, contractility, and vascular tone to preserve organ perfusion (53). Chronic catecholamine excess, however, promotes adverse remodeling by increasing myocardial oxygen demand, inducing myocyte apoptosis, stimulating fibroblast activation and extracellular matrix deposition, and driving pathological hypertrophy (54).

In the failing human heart, β-adrenergic receptors account for approximately 90% of adrenergic receptors and signal through G protein–adenylyl cyclase–cAMP–protein kinase A pathways to increase intracellular Ca²^+^ (55–57). Pediatric studies demonstrate developmental differences in β-adrenergic signaling (58). In adult heart failure, β1-adrenergic receptor expression is selectively downregulated, whereas pediatric idiopathic DCM is characterized by downregulation of both β1- and β2-adrenergic receptor transcripts, implying distinct adaptive and maladaptive responses across the life course (59).

Natriuretic peptides, particularly atrial natriuretic peptide (ANP) and B-type natriuretic peptide (BNP), counteract RAAS and sympathetic activation by inducing vasodilation, natriuresis, and direct antifibrotic and antihypertrophic effects (60). Pediatric cohorts show robust correlations between BNP or NT-proBNP levels and clinical heart failure scores, as well as prognostic associations with outcomes (61, 62).

### 2.2 Immuno-inflammatory remodeling

#### 2.2.1 Triggers and immune initiation

PHF often emerges at the intersection of myocardial stress and systemic inflammation (63, 64). Viral myocarditis, MIS-C, sepsis, and postoperative cardiopulmonary bypass can all provide potent immune triggers (65). In the injured myocardium, cardiomyocyte and endothelial stress releases damage-associated molecular patterns (DAMPs) that engage pattern-recognition receptors (PRRs) and amplify chemokine and cytokine signaling (66). This early innate sensing promotes leukocyte recruitment, microvascular dysfunction, and transient contractile depression; in susceptible hosts it can also seed persistent inflammation that drives remodeling (67, 68).

#### 2.2.2 Innate immunity, complement, and endothelial activation

Innate immune cells are major determinants of early phenotype severity (69). Neutrophils and inflammatory monocytes can worsen injury through oxidative stress, protease release, and microvascular plugging, while monocyte–macrophage programs shape the transition from inflammation to repair (70, 71). Complement activation and endothelial dysfunction further link systemic inflammation to myocardial edema and impaired perfusion, creating a feed-forward loop of hypoxia, mitochondrial stress, and reduced contractile reserve (72). Age-related differences in innate training and endothelial reactivity may contribute to the variable presentation of inflammatory cardiomyopathies in children (65).

#### 2.2.3 Adaptive immunity and loss of tolerance

Adaptive immune responses can sustain injury when antigen persists or immune regulation fails (69). T-cell polarization toward pro-inflammatory effector programs, inadequate regulatory T-cell control, and B-cell activation with pathogenic autoantibodies have all been implicated in immune-mediated cardiomyopathy (73, 74). In children, immune maturation and distinct exposure history may shape the threshold for tolerance breakdown and the likelihood of post-infectious autoimmunity (75).

Emerging evidence from Mendelian randomization (MR) studies has strengthened the causal link between specific immune cell phenotypes and heart failure susceptibility (76, 77). A recent MR analysis demonstrated that several immune cell traits are genetically associated with increased risk of heart failure, including CD66b++ myeloid cell AC, HLA DR on CD14- CD16 + monocyte, CD4 on CD4+, etc., a total of ten immune cell phenotypes (76). These findings provide genetic evidence implicating both innate and adaptive immune compartments—particularly myeloid cells, monocytes, B lymphocytes, and T lymphocyte subsets—in the pathogenesis of heart failure. Clinically, this biology supports an emphasis on phenotype-aware surveillance for persistent inflammation, arrhythmias, and late ventricular remodeling after apparent clinical recovery. Moreover, the identification of genetically supported immune cell subtypes offers potential targets for future precision immunomodulatory strategies in pediatric heart failure.

#### 2.2.4 Crosstalk with neurohormonal and stromal programs

Immune signaling does not act in isolation (78). Cytokines and complement fragments can potentiate sympathetic activation, alter beta-adrenergic responsiveness, and impair calcium handling, thereby intersecting with classic neurohormonal pathways (79, 80). Concurrently, immune cell–fibroblast crosstalk promotes extracellular matrix deposition and scar formation via profibrotic mediators and growth factors (81). This convergence provides a mechanistic bridge between acute inflammatory decompensation and chronic remodeling, and it motivates integrated management strategies that address both hemodynamic stress and immune activation (67, 79).

#### 2.2.5 Temporal dynamics and therapeutic windows

A clinically useful immune framework for PHF separates (i) an early inflammatory phase, in which immune activation may be adaptive but can become injurious, from (ii) a remodeling phase, in which incomplete resolution and maladaptive repair predominate (69). This distinction matters because immunomodulation is most plausible when an immune-mediated driver is active (e.g., myocarditis or MIS-C) and less likely to help once fixed remodeling dominates (65, 82). Longitudinal phenotyping—integrating symptoms, hemodynamics, biomarkers, and imaging trajectories—helps identify ongoing immune activity, define response, and guide escalation or de-escalation alongside standard heart failure therapy (82, 83). Understanding that immune contexts are dynamic rather than static is essential for clinical application. A single patient may transition through multiple contexts depending on intercurrent events. For example, a child with repaired congenital heart disease (the fixed etiology) may exhibit the expected postoperative systemic inflammation in the days following cardiopulmonary bypass. If that same child later develops a nosocomial infection, the context shifts to infection-associated decompensation, requiring a fundamentally different therapeutic approach focused on antimicrobial therapy and source control rather than immunomodulation. Conversely, a patient with stable genetic dilated cardiomyopathy may present with acute chest pain and troponin elevation, revealing a superimposed active immune-mediated injury (e.g., viral myocarditis) that warrants consideration of immunomodulation alongside standard heart failure therapy. These transitions underscore the need for serial reassessment and phenotype-aligned management rather than fixed treatment algorithms based on etiology alone.

#### 2.2.6 Conceptual immuno-inflammatory cascade

**Figure 2:**
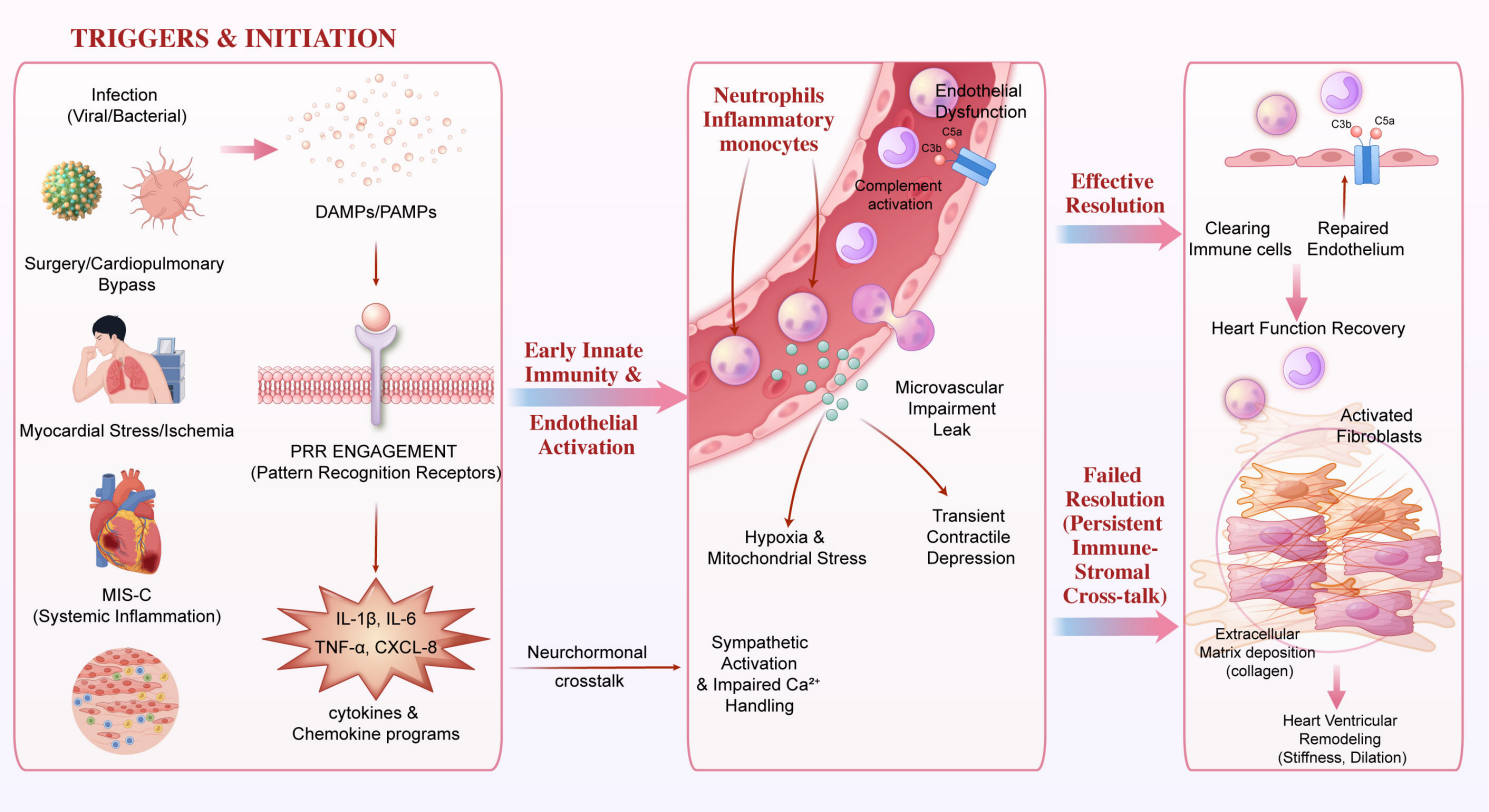
Immune–hemodynamic crosstalk driving remodeling.

Triggers such as infection, MIS-C, surgery/cardiopulmonary bypass, and myocardial stress generate pathogen- and damage-associated signals that engage pattern-recognition receptors, inducing cytokine and chemokine programs (66, 69). This promotes neutrophil and monocyte recruitment, complement activation, endothelial dysfunction, and microvascular impairment, which can precipitate contractile depression and electrical instability (84). With effective resolution, function may recover; with failed resolution, persistent immune-stromal crosstalk drives fibroblast activation, extracellular matrix deposition, and ventricular remodeling (69). The practical implication is to interpret biomarkers and imaging as trajectories—seeking concordant evidence of active immune injury versus predominantly remodeled disease—to time immunomodulation and intensity of surveillance (82). This cascade is illustrated in **Figure 2**.

### 2.3 Fibrosis and myocardial remodeling

Myocardial fibrosis is a key determinant of adverse outcomes in adult non-ischemic DCM, where late gadolinium enhancement on CMR strongly predicts mortality and sudden cardiac death (85). In contrast, studies of single-ventricle physiology suggest that fibrosis may be a secondary process in pediatric right ventricular failure, possibly reflecting shorter disease duration and different patterns of ventricular loading (86). These differences suggest that fibrotic remodeling may play a less central, or at least temporally distinct, role in pediatric versus adult heart failure.

### 2.4 MicroRNAs and gene expression

MicroRNAs (miRNAs) regulate post-transcriptional gene expression and play important roles in cardiac remodeling and heart failure (87, 88). In pediatric DCM, specific circulating miRNA profiles distinguish patients who recover ventricular function from those who progress to transplantation or death (89). Upregulation of miR-155 and miR-636 with downregulation of miR-646 and miR-639 is associated with adverse outcomes and may have prognostic value (89). Notably, canonical heart failure–related miRNAs in adults (e.g., miR-133a/b, miR-1, miR-21, miR-30, miR-23, miR-29) are often unchanged in children, underscoring age-dependent biology (90). These molecular differences may partly explain the relatively favorable pediatric response to certain inotropes and phosphodiesterase inhibitors compared with adults (90).

Transcriptomic analyses further support a distinct pediatric heart failure landscape (91). Tatman et al. identified genes differentially expressed in pediatric DCM that are not altered in adult DCM and observed attenuated cardiomyocyte hypertrophy in children compared with adults (91, 92). Collectively, these findings highlight fundamental developmental differences in heart failure pathobiology that should inform therapeutic strategies.

## 3 Diagnosis and phenotype-guided assessment

### 3.1 Biomarkers

In routine practice, evaluation can be anchored by a minimum bundle: (i) a natriuretic peptide (BNP or NT-proBNP), (ii) cardiac troponin, and (iii) echocardiography (65). When an inflammatory trigger is suspected, add CRP (and ferritin or IL-6 where available) to provide context rather than stand-alone diagnosis (93). For follow-up, trajectories and concordance across markers are generally more informative than isolated values, particularly in infants and in renal dysfunction (94).

#### 3.1.1 Conventional cardiac biomarkers

BNP and NT-proBNP are central to the diagnosis, risk stratification, and prognostic assessment of PHF, with superior diagnostic performance compared with many other biomarkers (95). BNP is synthesized primarily by ventricular myocytes in response to increased wall stress and acts as a functional marker of cardiac load and neurohormonal activation (96). However, BNP levels can be influenced by renal function, age, and the use of neprilysin inhibitors (97).

NT-proBNP, an inactive cleavage product released in equimolar amounts with BNP, has a longer half-life, lower biological variability, and higher analytical stability, making it particularly useful in children (98). NT-proBNP levels correlate with heart failure severity in infants and young children with CHD or DCM and serve as independent predictors of prognosis (61). Age- and sex-dependent reference ranges have been described, with higher values in early childhood and modestly higher levels in females from late adolescence onward (99). Elevated NT-proBNP primarily reflects increased myocardial wall stress (pressure/volume overload) and neurohormonal activation; values are strongly age-dependent and can be influenced by renal function (98). In clinical practice, serial trends are often more informative than single measurements, and higher concentrations are associated with more severe cardiac dysfunction and poorer treatment response (100).

Cardiac troponins, particularly cTnI and cTnT, are highly specific markers of myocardial injury (101). In heart failure, inflammatory cytokines (e.g., IL-1β, IL-6, TNF-α) and cell membrane damage facilitate troponin release into the circulation (102). Elevated troponin I is associated with worse outcomes in adult heart failure and coronary artery disease and provides incremental prognostic information when combined with natriuretic peptides (103). In pediatric settings, systematic assessment of cTnI in children presenting with chest pain improves detection of previously unrecognized cardiac disease and helps identify PHF exacerbations; combined measurement with NT-proBNP enhances diagnostic accuracy (104).

However, interpretation of troponin in children requires caution. Levels can be elevated in non-cardiac conditions such as severe infections, rheumatic diseases, and Kawasaki disease, and cut-offs vary with age and assay platform. While some studies have proposed pediatric reference intervals for high-sensitivity cTnT and cTnI, standardized diagnostic thresholds for PHF are still lacking (105). This represents a key gap that future multicenter studies should address.

##### 3.1.1.1 Novel cardiac biomarkers

Several emerging biomarkers show promise in PHF. Soluble ST2 (sST2), a member of the interleukin-1 receptor family, reflects myocardial stress and fibrosis and is less influenced by renal function and body mass index than natriuretic peptides; however, pediatric interpretation requires age- and sex-aware reference ranges (106, 107). Elevated sST2 levels correlate with adverse outcomes in adult heart failure and may have diagnostic and prognostic value in children, although pediatric data remain limited (108, 109).

Heart-type fatty acid–binding protein (H-FABP), a cytosolic protein released early in myocardial ischemia, can support diagnosis in patients with suspected acute coronary syndrome and negative troponin (110). In children with chronic heart failure, sST2 and H-FABP are significantly elevated, correlate with NT-proBNP, and appear independent of underlying etiology, suggesting potential roles in disease monitoring and risk stratification (109).

Galectin-3, a lectin implicated in fibrosis and macrophage activation, has shown associations with heart failure severity and adverse outcomes in small pediatric cohorts (109, 111, 112). Emerging evidence highlights its critical role in myocardial remodeling, as a key indicator of fibrosis and inflammation, positioning it as a promising biomarker for advancing heart failure diagnosis and management, particularly in congenital heart disease (113). Its relevance is especially pronounced in pediatric care, where early detection and intervention can substantially alter disease progression and improve outcomes (111). However, reported thresholds vary considerably by age, etiology, and assay platform, underscoring the urgent need for multicenter validation before clinical implementation (114). Cardiac myosin-binding protein C (cMyBP-C) is released with myocardial injury and has been proposed as an early biomarker in acute pediatric myocardial damage; preliminary studies suggest strong diagnostic performance, yet evidence remains limited and platform-dependent (115).

##### 3.1.1.2 Inflammatory and fibrosis-related markers

Inflammation and fibrosis are key contributors to heart failure progression. Classical inflammatory markers (e.g., CRP, IL-6, TNF-α) have been associated with PHF severity (116). Several newer inflammation- and fibrosis-related indicators have also been evaluated (117).

Connective tissue growth factor (CTGF), a profibrotic peptide, is elevated in pediatric heart failure and correlates with disease severity and echocardiographic indices; however, evidence for diastolic dysfunction–specific prediction in children remains limited (118). In a single-center report, combining CTGF with NT-proBNP improved diagnostic specificity and positive predictive value, but this incremental value requires external validation (118). Homocysteine promotes myocardial fibrosis and stiffness via both direct myocardial toxicity and vascular effects (119). In children with congestive heart failure, homocysteine has been associated with disease severity, echocardiographic indices, and adverse outcomes; an exploratory cut-off around 8.1 μmol/L was suggested in one cohort, but it needs independent, age-stratified validation before clinical use (117).

Pentraxin-3 (PTX-3), an acute-phase protein, participates in myocardial fibrosis and remodeling and rises early in heart failure (120). PTX-3 levels correlate with NT-proBNP, LVEF, and functional class and may support diagnosis and functional staging in pediatric heart failure (121). Syndecan-4, a transmembrane proteoglycan expressed on cardiac fibroblasts, is associated with tissue regeneration, angiogenesis, and cell adhesion; its serum levels correlate positively with NT-proBNP and functional class and negatively with LVEF, suggesting utility as a novel marker of heart failure severity (121, 122).

In clinical practice, inflammatory and fibrosis-related biomarkers may be most informative when interpreted as part of a multi-marker panel rather than in isolation (123). Combining hemodynamic stress (BNP/NT-proBNP), myocardial injury (troponins), inflammation (e.g., CRP and cytokine surrogates, PTX-3), and remodeling (sST2, galectin-3) can support phenotyping, track trajectories, and identify children at risk of decompensation, particularly in myocarditis, post-operative inflammatory states, and device-associated inflammation (123).

A major barrier to wider adoption is the lack of pediatric-specific reference ranges, timing standards, and assay harmonization (123, 124). Future studies should prioritize age-adjusted thresholds and longitudinal sampling strategies, and test whether inflammation-targeted interventions translate into measurable improvements in biomarker profiles and outcomes.

##### 3.1.1.3 Energy metabolism and neuroendocrine markers

Energy metabolism is intimately linked to heart failure pathogenesis (125). Adiponectin (APN), an adipocyte-derived hormone with anti-inflammatory and insulin-sensitizing properties, correlates with PHF severity in children with concomitant pneumonia (126). When combined with NT-proBNP, APN improves diagnostic sensitivity for PHF with pulmonary infection.

Neuroendocrine activation also plays a key role (127). Vasopressin contributes to fluid retention and vasoconstriction, but direct measurement is technically difficult (128). Copeptin, the C-terminal segment of pre-provasopressin, is more stable and serves as a surrogate marker of vasopressin secretion (129). Elevated copeptin in PHF correlates with disease severity and adverse events in children with cardiomyopathy-related heart failure, supporting its potential role in diagnosis and risk stratification (62).

Overall, multimarker strategies that combine natriuretic peptides, troponins, fibrosis markers, inflammatory mediators, and neurohormonal indices are likely to provide the most robust assessment of pediatric heart failure status and prognosis.

##### 3.1.1.4 Pragmatic inflammatory–immune phenotyping and trajectory-based monitoring (proposed)

In routine care, the goal is not deep immunophenotyping (65). Rather, clinicians need a feasible way to capture the inflammatory–immune context that changes management—raising or lowering suspicion for immune-mediated injury, tracking response to therapy, and flagging complications such as secondary infection (93).

To translate the conceptual phenotypes illustrated in **Figure 1** into clinical practice, **Table 1** proposes operational minimum criteria for each context. These criteria are designed to standardize phenotype assignment across different centers by specifying typical triggers, required bedside data points, and supportive findings.

**Table 1:** Operational minimum criteria for inflammatory-immune phenotypes in pediatric heart failure.

Phenotype	Typical triggers	Required data points (core bundle)	Supportive findings (enhanced bundle)	Time course	Entry criteria	Sources
Active Immune-Mediated Injury (Myocarditis, MIS-C)	Viral prodrome, SARS-CoV-2 exposure, vaccination	• Troponin >99th percentile • New ECG abnormalities (ST/T changes, conduction delay, arrhythmias) • Ventricular dysfunction (LVEF <50% or abnormal strain) • Inflammatory marker elevation (CRP, ESR)	• CMR meeting Lake Louise criteria (edema, LGE) • Fever ≥3 days (MIS-C) • Multisystem involvement (MIS-C) • Inflammatory cellular infiltrate on biopsy (lymphocytes) • Elevated IL-6, ferritin, D-dimer	Acute onset (days to 2 weeks)	All required criteria must be met	(7, 65, 130–134)
Postoperative Systemic Inflammation	Cardiopulmonary bypass, cardiac surgery	• Onset within 24–72 hours post-bypass • Fever (≥38.0 °C) or hypothermia • Vasoplegia or increased vasoactive-inotropic score (VIS) • CRP elevation	• Negative blood cultures • Rapid defervescence with expectant management • Elevated IL-6, IL-8, TNF-α • PTX-3 elevation • Absence of alternative infectious source	Acute (hours to days post-surgery)	All required criteria must be met; infection must be excluded	(135–137)
Infection-Associated Decompensation	Confirmed or suspected bacterial/viral infection	• Confirmed infection (positive culture, PCR, or clear clinical focus) • Acute deterioration in stable or compensated HF • BNP elevation • Hemodynamic instability (tachycardia, hypotension, poor perfusion)	• Positive microbiologic studies • Localized infection source • Response to antimicrobial therapy • Elevated PCT, lactate • Transient inflammatory marker elevation	Acute (hours to days)	First 2 criteria mandatory; at least 1 additional criterion	(138–140)
Chronic Remodeling with Low-Grade Inflammation	Genetic cardiomyopathy, complex CHD, post-inflammatory sequelae	• Chronic HF symptoms (>3 months) • Evidence of fibrosis/remodeling on imaging • Stable or slowly progressive course • Mildly elevated inflammatory markers (if any)	• CMR LGE or abnormal T1 mapping • Neurohormonal activation (BNP/NT-proBNP) • Fibrosis biomarkers (sST2, galectin-3) • Absence of acute trigger • Endomyocardial biopsy showing inflammation without acute necrosis	Chronic (months to years)	First 3 criteria required; inflammatory markers not mandatory	(1, 141–144)

Building upon this diagnostic framework, a practical approach to monitoring involves starting with a core bedside bundle that can be obtained rapidly and repeated, then adding decision-relevant markers when available (65). To ensure broad applicability, we propose a two-tiered phenotyping approach. The Core Bundle—basic labs, ECG, and echocardiography—is sufficient for initial phenotype assignment and monitoring in most settings. The Enhanced Bundle—advanced imaging (CMR with mapping), cytokine panels, and specialized biomarkers (sST2, galectin-3, PTX-3)—is reserved for tertiary centers or cases where core findings are inconclusive and results would alter management. During the diagnostic process, a key diagnostic challenge is distinguishing primary immune-mediated injury from secondary inflammatory markers arising from congestion or low output. This distinction rests on three pillars: (i) biomarker response to decongestion, (ii) imaging correlates of inflammation versus hemodynamic stress, and (iii) multi-domain concordance. Additionally, interpretation should emphasize trajectories (e.g., at presentation and after 24–48 hours, then as clinically indicated) rather than isolated values (145).

###### 3.1.1.4.1 Reassessment windows

To standardize this approach, we propose the following reassessment windows based on clinical context:

- Acute/fulminant presentations (e.g., suspected myocarditis, MIS-C with shock, postoperative deterioration): Reassess at 6–12 hours, then daily during ICU stay.
- Subacute presentations (e.g., newly diagnosed cardiomyopathy with stable hemodynamics): Reassess at 24–48 hours, then weekly or at key clinical milestones.
- Chronic ambulatory monitoring: Reassess at each clinical encounter (typically 1–3 months), with formal trajectory review every 6 months or at times of clinical change.
- Post-intervention monitoring: Reassess at 24–48 hours after clinical intervention (initiating immunomodulation or significant therapy escalation).

###### 3.1.1.4.2 Meaningful changes

A clinically significant shift is defined by either (i) an absolute numerical change in a key biomarker exceeding minimal clinically important difference, or (ii) concordant directional changes across multiple domains. Based on available pediatric data and expert consensus, we propose following provisional thresholds (7, 93, 132, 137, 146, 147):

- NT-proBNP/BNP: A change ≥30% from baseline (or ≥2-fold increase if no baseline available) is considered significant. In infants, higher thresholds (≥40%) may be appropriate due to greater physiological variability.
- Troponin: A doubling or halving from the previous value, or a change exceeding the 99th percentile upper reference limit, constitutes a meaningful shift.
- CRP: An absolute change ≥5 mg/dL or a relative change ≥50% from the previous value.
- Multi-domain concordance: A clinically significant trajectory is confirmed when at least two of the following domains show directional concordance: (a) hemodynamics (vasoactive-inotropic score change ≥5 points or lactate clearance <10% over 6 hours); (b) biomarkers (as defined above); (c) echocardiography (LVEF change ≥10 percentage points or strain improvement ≥15%); and (d) clinical status (improvement or deterioration in symptom scores, perfusion, or end-organ function).

###### 3.1.1.4.3 Practical assessment approaches

- Suspected myocarditis or MIS-C:
  • Core bundle: NT-proBNP/BNP + troponin + CRP (preferred combination); ECG; echocardiography with strain assessment when available (93).
  • Enhanced bundle (when clinically indicated and available): Ferritin; D-dimer (systemic hyperinflammation); IL-6 or other cytokines (selected cases); sST2; PCT (to assess bacterial co-infection) (93).
  • Action: Pair biomarker kinetics with serial echocardiography and clinical trajectory; consider cardiac MRI with T1/T2 mapping and late gadolinium enhancement for convalescent risk stratification when feasible (93).
- Postoperative systemic inflammation (e.g., after cardiopulmonary bypass):
  • Core bundle: NT-proBNP/BNP + lactate + CRP and/or PCT (preferred combination); clinical assessment of perfusion and vasoactive requirements (148).
  • Enhanced bundle (where available): IL-6 and/or PTX-3; echocardiographic strain imaging to detect subclinical ventricular dysfunction (149).
  • Action: Interpret early peaks in the context of timing after bypass, perfusion status, and evolving ventricular function; avoid over-calling myocarditis without supportive context (150).
- Sepsis/infection-associated decompensation:
  • Core bundle: NT-proBNP/BNP + troponin + lactate + CRP + PCT (preferred combination); blood cultures; source evaluation (151).
  • Enhanced bundle: Serial cytokine monitoring; echocardiographic parameters; consideration of myocardial tissue characterization if recovery is delayed or atypical.
  • Action: Prioritize source control, hemodynamic optimization, and oxygen delivery; do not over-attribute biomarker elevation to primary myocarditis unless supported by imaging/ECG features and exposure history.

###### 3.1.1.4.4 Key interpretation notes for trajectory monitoring

- Use age- and assay-appropriate reference ranges and avoid applying adult cutoffs to infants and young children;
- Standardize timing when possible (e.g., pre-treatment baseline and a 24–48 h reassessment) because early peaks may reflect stress physiology rather than ongoing immune injury.
- Integrate concordance across domains (clinical course, ECG, echo/strain, and biomarkers) instead of relying on any single marker.
- When inflammatory markers rise with worsening perfusion, reassess for infection, inadequate source control, or device-related complications before escalating immunomodulation.
- To differentiate primary immune injury from secondary signals:
  • Initiate decongestive therapy and reassess biomarkers at 24–48 hours. A ≥30% NT-proBNP reduction with clinical improvement suggests secondary inflammation; persistent elevation favors primary injury.
  • Disproportionate troponin elevation relative to hemodynamic compromise suggests primary myocardial inflammation.
  • Echocardiographic edema or CMR findings (T2 elevation, subepicardial LGE) support active immune injury.
  • Multi-domain concordance (biomarkers, imaging, ECG, clinical features) strengthens diagnostic confidence.
- In resource-limited settings, the core bundle (NT-proBNP/BNP, troponin, CRP, ECG, echocardiography) suffices for initial phenotype assignment and serial monitoring; enhanced modalities are not essential. Referral to a tertiary center is recommended when diagnostic uncertainty persists despite complete core evaluation, the patient deteriorates or fails to respond to therapy, advanced therapies are being considered, or a rare inflammatory phenotype is suspected. Telemedicine consultation can aid management when transfer is not feasible.

**Figure 3:**
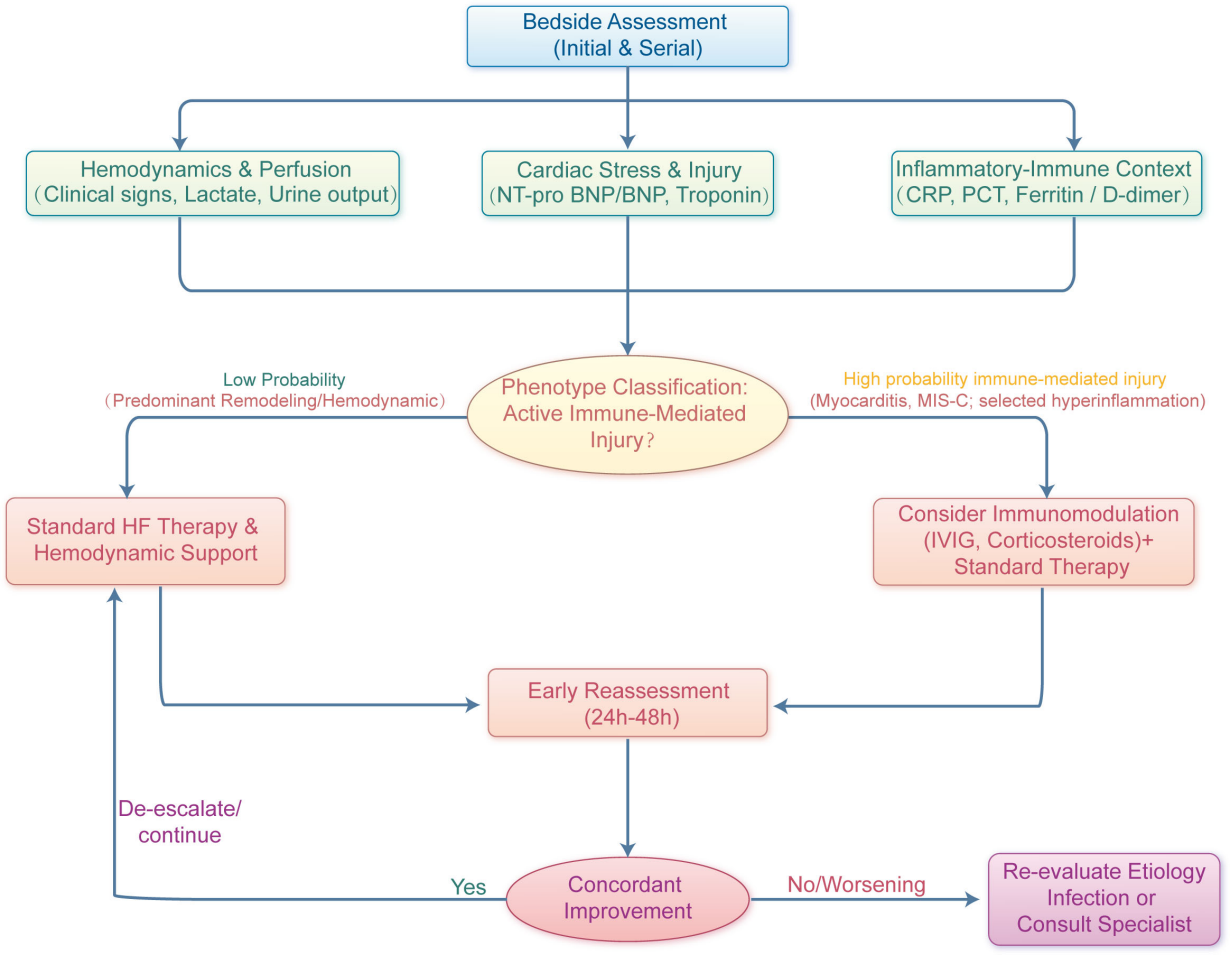
Bedside phenotype-guided assessment and treatment algorithm.

The integrated assessment workflow is summarized in **Figure 3**.

### 3.2 Imaging

#### 3.2.1 Echocardiography

Echocardiography is the first-line imaging modality in PHF due to its availability, portability, and ability to assess anatomy, function, and hemodynamics in real time (152). LVEF is widely used for systolic function, but pediatric cutoffs vary by age, loading conditions, and measurement method; where possible, interpret systolic function using age-adjusted norms (including z-scores when available) and complementary measures such as strain (153). Echocardiography should also systematically assess diastolic function, right ventricular performance, pulmonary pressures, and lesion-specific physiology, as these often drive symptoms and management more than LVEF alone (152). Serial studies are most informative when obtained with consistent technique and in relation to clinical events (therapy changes, infection, postoperative transitions) (154).

Contemporary pediatric echocardiography guidance supports body-size normalization (e.g., BSA-adjusted Z-scores) for many measurements (153). However, diastolic dysfunction criteria remain less standardized in children, and Z-score–based algorithms have been proposed to aid earlier detection compared with fixed absolute cut-offs (155). Speckle-tracking echocardiography provides sensitive measures of myocardial deformation, such as global longitudinal strain, which can detect subclinical systolic dysfunction before LVEF declines (156). Incorporating these advanced parameters may facilitate earlier intervention in high-risk pediatric patients.

#### 3.2.2 Chest radiography, electrocardiography, and ambulatory ECG

Chest radiography remains a useful adjunct for demonstrating cardiomegaly, pulmonary vascular congestion, and pleural effusions, which may precede overt clinical decompensation and help guide early intervention (157).

Standard 12-lead electrocardiography (ECG) does not diagnose heart failure per se but can reveal structural and electrical abnormalities associated with higher risk (158). In children with structural heart disease and heart failure, prolonged corrected QT interval and QRS duration correlate with ventricular dysfunction and predict mortality or transplantation (159). Twenty-four-hour ambulatory ECG (Holter) can detect occult arrhythmias, heart rate variability abnormalities, and repolarization disturbances (160). Parameters derived from Holter monitoring, including arrhythmic burden and reduced heart rate variability, independently predict progression and mortality in heart failure (160).

#### 3.2.3 Cardiac magnetic resonance imaging

CMR provides quantitative assessment of ventricular volumes, mass, and function, and offers unparalleled tissue characterization (161). In children with heart failure, CMR aids in etiologic diagnosis (e.g., myocarditis, cardiomyopathies, infiltrative diseases), differentiates ischemic from non-ischemic injury, and quantifies fibrosis and edema (65, 161). Techniques such as late gadolinium enhancement, T1 and T2 mapping, and extracellular volume fraction estimation are particularly informative (162, 163).

Limitations include long scan times, need for breath-holding or sedation, and susceptibility to motion artifacts, which constrain use in unstable or very young children (164). Nevertheless, when feasible, CMR plays an increasingly central role in phenotyping and risk stratification in PHF.

### 3.3 Combined assessment and clinical prediction models

Multimodal diagnostic approaches outperform single-parameter assessments. Combining echocardiography with NT-proBNP, growth differentiation factor-15, troponins, and other biomarkers improves sensitivity and specificity for PHF diagnosis and severity grading compared with isolated measurements (165, 166). Integration of echocardiography, cardiovascular computed tomography, and CMR can significantly enhance etiologic diagnosis, especially in complex congenital heart disease and cardiomyopathy (167).

While numerous prediction models exist for adult heart failure, pediatric-specific tools remain scarce (105). An early warning score for acute heart failure in infants, incorporating oxygen saturation, urine output, heart rate, emotional state, and respiratory rate, achieved accuracies above 90% in validation using logistic regression and neural network models, but clinical uptake is limited (168). Other studies have identified risk factors such as CHD, abnormal myocardial enzymes, and need for moderate- to high-flow oxygen in children with severe pneumonia, but without constructing formal prediction models.

The Ibadan childhood heart failure score correlates strongly with plasma BNP (r = 0.920) and shows high sensitivity (97.6%) and specificity (89.8%) at a cutoff of 2 points, supporting its utility in low-resource settings for rapid diagnosis and severity assessment (169). Patel et al. developed a model predicting death or transplantation in neonates, children, and young adults using echocardiographic and laboratory variables (LVEF, lymphocyte count, serum sodium, bicarbonate, and creatinine), achieving 82.1% accuracy (170). Additional work has focused on specific subgroups such as children with malignancy-associated heart failure. A hospitalization heart failure score has also been proposed to quantify symptom severity (respiratory distress, feeding difficulty, reduced activity), but multicenter validation is pending (171).

Overall, high-quality, multicenter studies are needed to develop and validate PHF-specific prediction models across diverse etiologies and healthcare settings.

### 3.4 Functional classification and staging

Accurate functional classification and staging of heart failure are central to therapeutic planning and prognostic assessment (172, 173). In children, the New York Heart Association (NYHA) classification and the modified Ross classification are most commonly used and are endorsed by major guidelines (174, 175). The modified Ross score adapts symptom descriptors for infants and young children (e.g., feeding difficulty, growth failure) and provides a more age-appropriate assessment of functional limitation (174).

Staging concepts originally proposed for adults by the American Heart Association—Stage A (at risk), Stage B (structural heart disease without symptoms), Stage C (symptomatic heart failure), and Stage D (refractory end-stage disease)—have now been adapted for children, including those with congenital heart disease (142, 173). National consensus documents emphasize the importance of stage-based management algorithms that incorporate prevention, early intervention, advanced therapies, and palliative care across the continuum of pediatric heart failure (142, 176).

## 4 Management

### 4.1 Etiology-directed therapy

Whenever the underlying cause of PHF can be identified and addressed, targeted therapy may substantially modify or even reverse the heart failure phenotype (27, 142). In children, structural congenital anomalies and inborn errors of metabolism account for a large share of etiologies, rendering a meaningful proportion of patients amenable to causative interventions (177).

#### 4.1.1 Genetic and metabolic disorders

Inborn errors of metabolism represent an important cause of pediatric cardiomyopathy and heart failure (178). Defects in glycolysis, amino acid and organic acid metabolism, fatty acid β-oxidation, lysosomal function, and mitochondrial bioenergetics can all lead to ventricular dysfunction (179). Early recognition is crucial because specific therapies may improve outcomes (178).

Pompe disease (glycogen storage disease type II) is an autosomal recessive disorder caused by deficiency of acid α-glucosidase, leading to lysosomal glycogen accumulation in skeletal muscle, myocardium, and other organs (180). Infantile-onset Pompe disease presents within the first year of life with progressive hypotonia, feeding difficulties, and cardiomegaly and is uniformly fatal within 12 months without treatment (181). Late-onset forms present later in childhood or adulthood with proximal myopathy and respiratory insufficiency, usually with less prominent cardiac involvement (180). Enzyme replacement therapy with recombinant human α-glucosidase improves survival and cardiac outcomes, especially when initiated early (182). In a 78-week randomized trial of 90 ambulatory patients aged ≥8 years with late-onset Pompe disease, alglucosidase alfa (20 mg/kg biweekly) significantly improved 6-minute walk distance (28.1+/-13.1 meters, p=0.03) and forced vital capacity (3.4+/-1.2%, p=0.006) versus placebo, with comparable safety (183).

Fabry disease, an X-linked lysosomal storage disorder caused by deficiency of α-galactosidase A, results in progressive accumulation of globotriaosylceramide and multiorgan involvement (184). Cardiac manifestations in children include valvular disease, conduction abnormalities, arrhythmias, and left ventricular hypertrophy (185). Enzyme replacement therapy with agalsidase alfa or beta reduces left ventricular mass, improves cardiac function and exercise tolerance, and slows renal disease progression, but these functional benefits occur only in Fabry patients without baseline myocardial fibrosis, with a 3-year study of 32 patients showing no functional improvement in those with pre-existing fibrosis despite modest hypertrophy reduction (186).

Barth syndrome is an X-linked mitochondrial disorder characterized by cardiomyopathy, neutropenia, skeletal myopathy, and growth delay (187). Mitochondria-targeted therapies have gained momentum; notably, elamipretide received U.S. FDA accelerated approval in 2025 as a treatment for Barth syndrome in adult and pediatric patients weighing at least 30 kg (188). This regulatory milestone reflects a high unmet need and signals of benefit, but it also underscores the importance of confirmatory outcome data and careful long-term safety surveillance in children (189, 190).

#### 4.1.2 Surgical management

##### 4.1.2.1 Pulmonary artery banding

Pulmonary artery banding is traditionally used as a palliative procedure in infants with large left-to-right shunts or complex congenital defects to control pulmonary overcirculation, reduce left ventricular volume overload, and improve heart failure symptoms (191, 192). More recently, its use has been explored in children with dilated cardiomyopathy and severe functional mitral regurgitation to promote reverse remodeling of the left ventricle, with some reports of improved function (193, 194).

##### 4.1.2.2 Septal myectomy

Hypertrophic cardiomyopathy (HCM), particularly when associated with dynamic left ventricular outflow tract obstruction, can cause severe heart failure and sudden cardiac death in children (195). For patients with symptomatic obstructive HCM refractory to medical therapy, surgical septal myectomy is the current standard of care to relieve obstruction and improve symptoms (196).

Compared with adults, septal myectomy in children is technically more challenging because of smaller aortic annuli and more complex anatomy, and the risk of residual or recurrent obstruction is higher (197, 198). In a series of 37 children undergoing transaortic septal myectomy (median age 7.4 years, nearly half with RASopathies), 20-year transplant-free survival was 80.6% and freedom from HCM-related mortality was 87.1%, but about one-third required reintervention (199). Overall, surgical management of obstructive HCM in children is associated with low perioperative mortality and good long-term outcomes, but requires lifelong follow-up.

Mechanistic studies using patient-specific induced pluripotent stem cell–derived cardiomyocytes have shown that MYH7 and biallelic MYBPC3 variants cause hypercontractility via enhanced myosin–actin interaction and increased ATPase activity, whereas monoallelic MYBPC3 variants produce milder hyperdynamic changes (200). Myosin ATPase inhibitors normalize structural and functional abnormalities across genotypes more comprehensively than conventional drugs, supporting genotype-guided precision therapy in pediatric HCM (200).

##### 4.1.2.3 Correction of structural anomalies

Surgical correction of structural cardiovascular anomalies underlying heart failure—such as anomalous coronary origins, complex CHD, or great vessel stenosis—can markedly improve cardiac function and prognosis (201). In children with Kawasaki disease, coronary artery bypass grafting may be indicated for giant aneurysms, ischemia, or heart failure and can significantly improve symptoms and long-term outcomes (202).

#### 4.1.3 Immune-mediated and inflammatory cardiomyopathies

##### 4.1.3.1 When to suspect an immune-mediated driver

Immune-mediated myocardial injury should be considered when heart failure onset is abrupt or disproportionate to structural disease, particularly after viral prodrome, systemic inflammation, or vaccination (65). Clues include fever, rapid troponin/BNP rise, new conduction abnormalities, myocardial edema on imaging, and systemic hyperinflammatory syndromes such as MIS-C (65, 203). When evaluating acute decompensation, clinicians must distinguish between a superimposed immune-mediated process (e.g., viral myocarditis) and progression of the underlying etiology (e.g., genetic cardiomyopathy). Clues favoring a superimposed process include abrupt onset, fever, disproportionate troponin elevation, and new ECG changes—features aligned with those listed above. Conversely, chronic, slowly progressive deterioration without these features suggests established remodeling unlikely to respond to immunomodulation (204). Importantly, these scenarios are not mutually exclusive: structural heart disease can host acute myocarditis, and post-inflammatory sequelae may become a new etiologic substrate. This dynamic relationship underscores the need for serial reassessment rather than fixed treatment algorithms based on etiology alone.

##### 4.1.3.2 Myocarditis—practical bedside framing

Clinicians should suspect myocarditis (MC) when acute or subacute heart failure follows a viral prodrome and is accompanied by disproportionate myocardial injury markers, ECG abnormalities/arrhythmias, or rapid hemodynamic deterioration (65). A pragmatic diagnostic package integrates BNP/NT−proBNP, troponin, CRP (and other inflammatory markers as clinically indicated), ECG, and serial echocardiography (including strain when available); CMR is most useful for tissue characterization and convalescent risk stratification when feasible (205). From a cardio−immunology perspective, the key bedside distinction is active immune−mediated injury with ongoing inflammatory signal versus predominantly remodeled disease with fixed dysfunction and low inflammatory activity (65). The evidence base for immunomodulation in pediatric MC varies by clinical presentation. Fulminant MC can be distinguished from acute MC clinically by features such as severe hemodynamic compromise and cardiogenic shock, and histologically by more extensive inflammatory infiltration compared to non-fulminant cases (206, 207). A comparative retrospective survey involving 169 pediatric patients with MC demonstrated that among those with acute MC, 1-month survival rates did not differ between patients receiving intravenous immunoglobulin (IVIG) (92.3%) and those receiving no such therapy (87.5%). In contrast, among patients with fulminant MC, survival tended to be higher in those treated with IVIG compared to untreated patients (78.6% vs. 0%). However, the differential efficacy of IVIG between two phenotypes did not reach statistical significance, and this study carries important limitations inherent to its non-randomized design (206). These findings underscore the importance of phenotype-tailored immunomodulatory approaches. Accordingly, management should prioritize hemodynamic stabilization and standard heart failure therapy in parallel; immunomodulation, when considered, should be phenotype−aligned and time−window–sensitive rather than triggered by nonspecific biomarker elevation (65). Given that recovery can be incomplete, longitudinal surveillance for residual inflammation, arrhythmia burden, and adverse remodeling is warranted.

##### 4.1.3.3 Immunomodulatory strategies: principles rather than recipes

Before initiating immunomodulation, a structured pre-treatment assessment reduces harm and improves the chance of benefit.

1. Before initiating immunomodulation, exclude congestion as the primary driver: optimize hemodynamics, document biomarker response to decongestion, and review imaging for inflammatory correlates. Immunomodulation is most justified when multi-domain findings align to suggest active immune injury.
2. Define the most likely immune context and probability of immune causality (e.g., fulminant lymphocytic myocarditis, MIS-C, immune checkpoint inhibitor–related myocarditis, post-infectious inflammatory phenotype) rather than treating nonspecific biomarker elevation (65).
3. Exclude or actively address uncontrolled infection: obtain cultures as appropriate, evaluate for bacterial sources, and interpret PCT/CRP and clinical findings together. Immunomodulation should generally not delay antibiotics or source control when infection is plausible (151).
4. Establish a baseline severity profile across domains—hemodynamics/perfusion, ECG/rhythm, echocardiography (including strain if available), key labs (BNP/NT-proBNP, troponin, CRP ± ferritin/D-dimer), and organ function—so that response and toxicity can be judged objectively (65).
5. Predefine response targets and stopping rules (e.g., improvement in vasoactive support, biomarkers, ventricular function) and plan an early reassessment (typically within 24–48 hours) (93).
6. Involve pediatric cardiology, infectious diseases, and critical care early for phenotype alignment and safety oversight, particularly when considering biologics (65).

Supportive heart failure therapy and hemodynamic stabilization remain foundational and should proceed in parallel (65). Immunomodulation, when considered, should be phenotype-aligned, risk-aware, and guided by the best available pediatric evidence and disease-specific guidance (e.g., myocarditis and MIS-C pathways) (208). IVIG and corticosteroids are commonly used in selected immune-mediated phenotypes, but benefits and optimal regimens vary by context and are not established for all PHF presentations (209). Targeted agents (e.g., anakinra or tocilizumab) may be considered for refractory hyperinflammation in carefully selected cytokine-driven phenotypes, ideally within institutional protocols and with specialist input (208). Across strategies, clinicians should (i) reassess early (24–48 hours) for objective improvement, (ii) avoid escalation in the setting of worsening or uncontrolled infection, and (iii) taper or stop promptly when inflammatory activity resolves or when toxicity and infection risk outweigh potential benefit (208).

##### 4.1.3.4 Immune-guided monitoring, de-escalation, and safety

Because pediatric evidence is heterogeneous, a conservative, trajectory-based approach is appropriate (65). Response should be tracked using a small set of objective domains:

- Hemodynamics and perfusion (including vasoactive requirements, lactate, urine output, and end-organ function) (151).
- Cardiac injury and stress (BNP/NT-proBNP and troponin trends, interpreted with age-adjusted norms) (210).
- Inflammatory–immune context (CRP/PCT ± ferritin/D-dimer/cytokines where available), with active surveillance for secondary infection (208).
- Cardiac structure and function (serial echocardiography; consider CMR in convalescence when feasible) (211).

**Figure 4:**
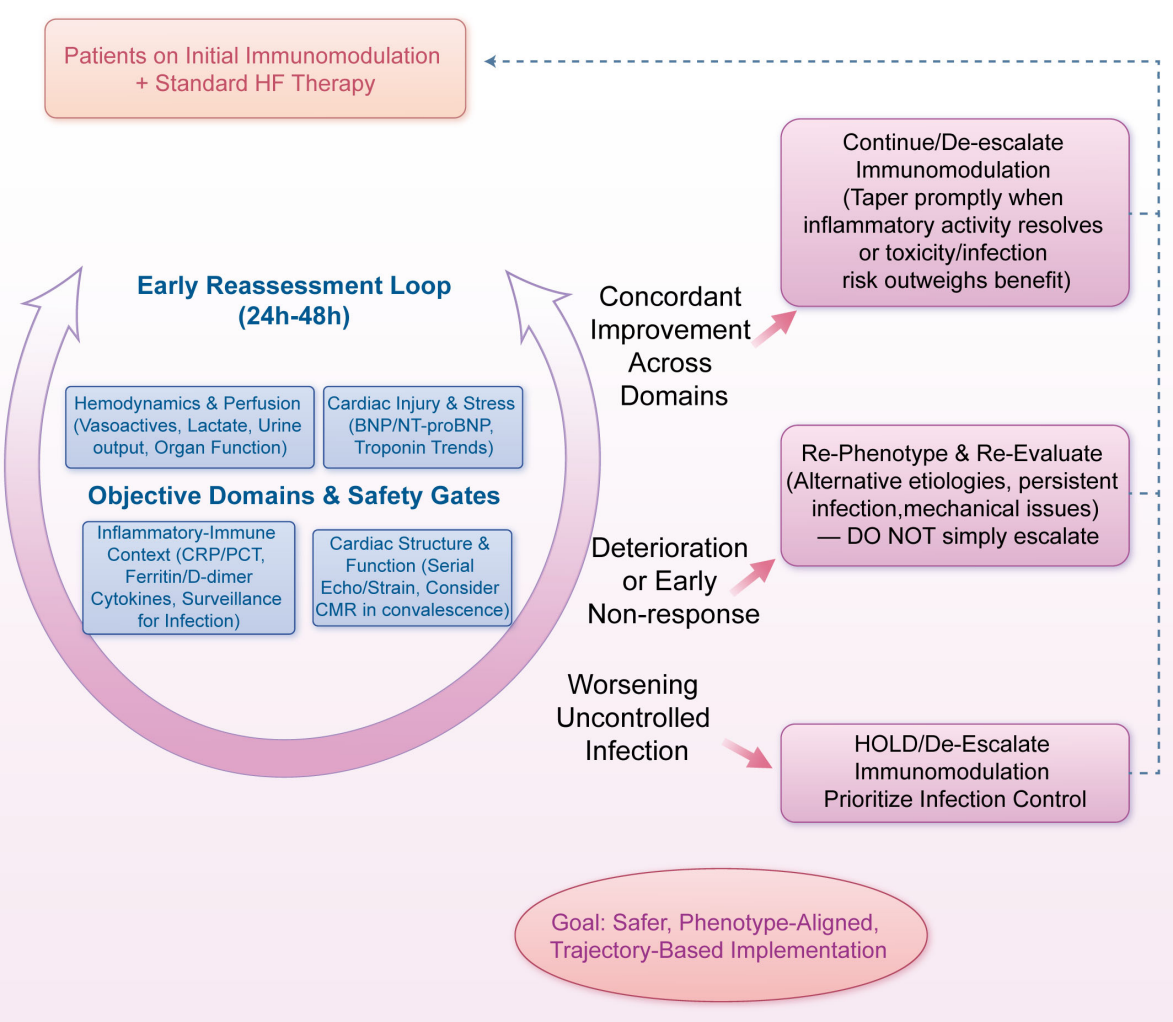
Immunomodulation safety gates and reassessment loop.

Escalate or de-escalate immunomodulation based on concordant improvement or deterioration across these domains rather than a single laboratory value (151). Early non-response should trigger re-phenotyping (e.g., alternative etiologies, persistent infection, mechanical issues) before simply intensifying immunosuppression (65). These safety gates and the early reassessment loop are summarized in **Figure 4**.

##### 4.1.3.5 MIS-C with cardiac involvement: practical points

MIS−C typically presents as systemic hyperinflammation with endothelial activation and capillary leak, and myocardial dysfunction can be prominent even without classic myocarditis symptoms (212). Bedside phenotyping should integrate BNP/NT−proBNP and troponin with CRP (and ferritin/D−dimer or cytokine markers where available) alongside echocardiography; CMR can be considered in convalescence to assess residual edema or scar when feasible (208). Treatment should address systemic inflammation in parallel with hemodynamic stabilization, with close monitoring for both rapid improvement and potential relapse or delayed remodeling (93). Post−acute surveillance should include rhythm assessment and follow−up evaluation for persistent biomarker elevation or subclinical ventricular dysfunction (213).

##### 4.1.5.6 Chronic heart failure in complex congenital heart disease

Children and young adults with complex congenital heart disease (CHD) may develop a distinct chronic heart failure syndrome despite technically successful repairs (214). Fontan circulation failure and systemic right ventricular (RV) failure illustrate a hemodynamic–immuno-metabolic phenotype characterized by chronically elevated venous pressures, lymphatic dysfunction, hepatic and intestinal congestion, and low-grade systemic inflammation (215). These features may amplify endothelial activation, cytokine signaling, and fibrosis, contributing to progressive exercise intolerance, arrhythmias, and end-organ dysfunction (216).

Management prioritizes careful phenotyping of the dominant failure mode (ventricular systolic dysfunction, diastolic limitation, atrioventricular valve regurgitation, pulmonary vascular disease, lymphatic failure, or arrhythmia-mediated decline) and targets reversible drivers (217). Standard neurohormonal blockade may be trialed for ventricular dysfunction, but benefit is variable (218). Aggressive rhythm surveillance and management, optimization of preload/afterload, treatment of pulmonary vascular disease where present, and timely referral for advanced therapies (VAD or transplantation) are crucial (219). Because these circulations have heightened thromboembolic and infection vulnerability, anticoagulation and infection vigilance should be individualized, and immunomodulation should be reserved for clearly defined inflammatory syndromes rather than chronic congestion-driven biomarker elevations alone (220).

### 4.2 Advances in pharmacotherapy

Multiple guidelines inform pharmacologic treatment of PHF, but robust pediatric evidence is still limited. As a result, many therapies are extrapolated from adult heart failure with careful dose adjustments and monitoring. Recent studies have begun to evaluate newer drug classes in children (48, 221). Notably, the International Society for Heart and Lung Transplantation (ISHLT) has recently proposed updated recommendations for the management of chronic heart failure in children, highlighting meaningful therapeutic advances. These include support for angiotensin receptor-neprilysin inhibitors (ARNIs), sodium-glucose cotransporter 2 inhibitors (SGLT2is), and soluble guanylate cyclase (sGC) stimulators in the treatment of pediatric chronic heart failure with reduced ejection fraction (HFrEF) (222).

#### 4.2.1 Angiotensin receptor–neprilysin inhibitors

Sacubitril/valsartan combines a neprilysin inhibitor (sacubitril) and an angiotensin II type 1 receptor blocker (valsartan) (223). Neprilysin inhibition augments endogenous natriuretic peptides, while RAAS blockade reduces vasoconstriction and remodeling (223). Based largely on adult data, the U.S. Food and Drug Administration approved sacubitril/valsartan in 2019 for symptomatic heart failure due to systemic left ventricular systolic dysfunction in children aged ≥1 year. According to the 2024 ISHLT guideline recommendations, ARNI is considered a reasonable alternative to angiotensin-converting enzyme inhibitors or angiotensin receptor blockers in pediatric patients older than one year of age with HFrEF (Class IIa, Level of Evidence B) (222).

PANORAMA-HF was a randomized, double-blind trial comparing sacubitril/valsartan with enalapril in children and adolescents with symptomatic heart failure due to systemic left ventricular systolic dysfunction (224). This single pediatric randomized controlled trial enrolled 375 children aged 1 month to <18 years with LVEF ≤45% or fractional shortening ≤22.5% (level of evidence: B). The primary endpoint was a global rank composite score, ranking patients from worst to best outcome based on clinical events including death, listing for urgent heart transplant, and others. Although no significant difference was observed between treatment groups (Mann-Whitney probability, 0.52 [95% CI, 0.47–0.58]; Mann-Whitney odds, 0.91 [95% CI, 0.72–1.14]; P = 0.42), both regimens improved symptoms and reduced NT-proBNP levels, with comparable safety and tolerability profiles (48). However, the study’s limitations include being underpowered for subgroup analyses and having a relatively short follow-up period (52 weeks) which precludes assessment of long-term outcomes. Accordingly, sacubitril/valsartan may be considered in selected pediatric patients, but superiority over ACE inhibition has not been established and longer-term outcome data remain needed.

#### 4.2.2 If channel inhibition

Tachycardia is common in pediatric heart failure and contributes to reduced diastolic filling time and increased myocardial oxygen demand (225). Ivabradine selectively inhibits the If current in the sinoatrial node, slowing heart rate without negative inotropic effects (226). A multicenter, randomized, double-blind trial involving 116 children aged 6 months to <18 years with DCM and symptomatic chronic heart failure demonstrated that ivabradine added to background therapy significantly reduced resting heart rate. The primary endpoint—a ≥20% reduction in resting heart rate without symptomatic bradycardia—was achieved in 70% of ivabradine-treated patients versus 12% of placebo recipients (odds ratio 17.24, 95% confidence interval 5.91–50.30, p < 0.0001). Ivabradine also improved LVEF (mean increase 13.5% vs. 6.9%, p = 0.024) and NYHA or Ross functional class at 12 months (level of evidence: B). However, it did not demonstrate a mortality benefit or composite endpoint effect, and its reliance on a heart rate reduction–based primary endpoint limits definitive conclusions regarding clinical efficacy (227).

A separate small retrospective series of 13 infants under 6 months of age treated with off-label ivabradine for heart failure or supraventricular tachycardia reported significant reductions in heart rate and improvements in LV function (evidence level: C); bradycardia occurred in three patients (23%), although only one required drug discontinuation (228). While these preliminary data suggest that ivabradine may be effective and safe in carefully selected neonates and infants, important limitations must be acknowledged. The pivotal pediatric trial excluded infants under 6 months and patients with acute decompensation, long-term safety data beyond 12 months remain lacking, and evidence in this youngest age group is confined to small case series (228). Larger prospective studies are therefore warranted to confirm these findings and establish safety.

#### 4.2.3 Sodium–glucose cotransporter 2 inhibitors

SGLT2 inhibitors have emerged as important therapies in adult heart failure irrespective of diabetes status and may confer benefits through natriuresis, osmotic diuresis, improved cardiac metabolism, and anti-inflammatory effects (229, 230). Pediatric experience remains limited but growing. The 2024 ISHLT guidelines classify SGLT2 inhibitors as a Class IIb recommendation (Level of Evidence C) for use in pediatric heart failure. Despite the limited evidence base, emerging studies support their safety and tolerability in children, suggesting potential as an adjunctive therapy warranting further investigation (222, 231).

An observational study of 49 patients (age 10–19 years, mean 14.5 years) with chronic heart failure treated with dapagliflozin 10 mg daily added to standard therapy (RAAS inhibitors, mineralocorticoid receptor antagonists, β-blockers) reported improved LVEF (from 35% to 40%, OR 1.04 (1.01–1.09), p = 0.02), trends toward lower NT-proBNP (from 2606 pg/ml to 1110 pg/ml, p = 0.07), and improved functional class over 6 months, without severe hypotension or renal impairment (prospective observational cohort, level of evidence C) (232). Genital candidiasis and sterile pyuria occurred in a minority of patients (232). These findings suggest that SGLT2 inhibitors may be a safe and effective adjunct in pediatric heart failure, but randomized trials are required (232). The limitations lie in: non-randomized design with potential selection bias; small sample size; short follow-up (6 months); no control group; heterogeneity of underlying etiologies; lack of pharmacokinetic data in younger children.

#### 4.2.4 Other heart failure agents

Recombinant human BNP (rhBNP) mimics endogenous BNP and exerts vasodilatory, natriuretic, and anti-remodeling effects without significant electrolyte disturbances (233). RhBNP (nesiritide) was evaluated in a prospective single-arm study involving 32 children aged 1 month to 17 years with acute decompensated heart failure (Level of evidence: C) (234). Nesiritide administration (0.01–0.03 μg/kg/min infusion) was associated with significant improvements in clinical and hemodynamic parameters: urine output increased from a baseline of 2.35 ± 1.71 mL/kg/hr to 3.10 ± 1.94 mL/kg/hr by day 4 (p < 0.01); mean central venous pressure decreased from 13 mmHg to 7 mmHg (p = 0.018); and mean NYHA functional class improved significantly (p < 0.001). However, the study was limited by its uncontrolled design, small sample size, lack of a comparator group, short follow-up duration, absence of standardized age-specific dosing, and lack of data on hard outcomes such as mortality or transplant-free survival.

Newer adult therapies, such as cardiac myosin activators and the soluble guanylate cyclase stimulator vericiguat, lack published data in the PHF population (235).However, ongoing systematic investigations are evaluating the efficacy of vericiguat in children with stabilized systolic heart failure, positioning it as a promising candidate for future pediatric heart failure pharmacotherapy (222, 236). Iron deficiency is common in chronic heart failure and is associated with reduced exercise capacity and worse prognosis; intravenous iron improves symptoms and quality of life in adult heart failure, but pediatric data are sparse (237, 238). Periodic assessment of iron status and individualized replacement strategies may be reasonable in children with PHF, especially in the context of reduced intake or chronic inflammation (239).

Pediatric drug development faces unique challenges, including small sample sizes, age-dependent pharmacokinetics and pharmacodynamics, ethical constraints, and heterogeneity of underlying disease (240). High-quality pediatric trials are therefore urgently needed to refine dosing, assess long-term safety, and define optimal therapeutic combinations.

### 4.3 Cellular therapy

In adults with chronic heart failure, intracoronary or intramyocardial delivery of bone marrow-derived cells has shown modest improvements in ventricular function and quality of life in selected studies (241). Pediatric experience is limited to case reports and small series, but early results suggest that cell therapy may be feasible and potentially beneficial in refractory pediatric cardiomyopathy (242). However, heterogeneity in cell type, delivery route, dosing, and outcome measures, coupled with a lack of long-term follow-up, precludes definitive conclusions at present.

### 4.4 Device therapy

CRT, mechanical circulatory support (ECMO and VADs), and novel shunt devices have expanded the therapeutic armamentarium for PHF (243, 244).

CRT is well established in adult heart failure with reduced ejection fraction, but pediatric evidence is largely observational and heterogeneous because substrates differ (e.g., congenital heart disease, systemic right ventricle, postoperative conduction disease) (245, 246). A single-center retrospective comparative study using propensity score-matched analysis identified 63 matched pairs of CRT patients and controls, in which CRT patients were congenital heart disease or age <21 years who had symptomatic heart failure, systemic ventricular ejection fraction <45%, evidence of electrical dyssynchrony, and then underwent CRT implantation (247). This registry-based analysis demonstrated that CRT was associated with significantly improved transplant-free survival, with a relative risk reduction for death or heart transplantation (hazard ratio 0.24; 95% confidence interval 0.12 to 0.46; P<0.001) (level of evidence: C). Nevertheless, these findings should be interpreted in light of several limitations inherent to the registry design, including potential selection bias, heterogeneous patient populations, variability in implantation techniques and lead placement, and absence of standardized outcome definitions. Additionally, nonresponse remains common, emphasizing the importance of meticulous electrical and anatomic characterization and careful patient selection.

For children with refractory heart failure despite maximal medical therapy, ECMO or VAD implantation can provide life-saving support as a bridge to recovery, transplantation, or decision (248). ECMO use has expanded in pediatric centers, particularly after cardiac surgery and in fulminant myocarditis, where survival is relatively favorable (249). VADs may offer better long-term stability and improved transplant outcomes compared with ECMO in selected children, especially those with chronic cardiomyopathy (244).

Atrial septostomy and dedicated intracardiac shunt devices (e.g., atrial flow regulators) have been explored to reduce left atrial pressure and pulmonary congestion in advanced heart failure (250). Early reports in small pediatric series demonstrate improved oxygen saturation, functional class, and symptomatic relief, with acceptable short-term safety, but larger studies are required (251).

### 4.5 Heart transplantation

#### 4.5.1 Transplant immunobiology and rejection phenotypes

Pediatric heart transplantation has evolved over nearly five decades and remains the definitive therapy for selected children with end-stage heart failure (252). From an immunologic perspective, long-term outcomes are shaped by the balance between preventing rejection and minimizing infection, malignancy, and metabolic toxicity (252). Cellular-mediated rejection and antibody-mediated rejection reflect distinct mechanisms and may require different diagnostic and therapeutic approaches.

ABO-incompatible transplantation has expanded the infant donor pool by leveraging developmental immunology (253). In early life, low isohemagglutinin titers and evolving humoral immunity can permit successful ABO-incompatible graft acceptance when carefully selected and monitored (253). This strategy can reduce waiting-list mortality in the highest-risk age group while maintaining acceptable post-transplant outcomes (254).

#### 4.5.2 Immune surveillance, individualized immunosuppression, and late complications

Advances in perioperative care, immunosuppressive regimens, and post-transplant surveillance have steadily improved survival (255). However, late complications—especially cardiac allograft vasculopathy, chronic rejection, and infection—remain major determinants of long-term morbidity (256). Practical surveillance integrates clinical assessment, echocardiography, endomyocardial biopsy where appropriate, and serologic monitoring (including donor-specific antibodies when available), with risk stratification based on age, sensitization history, adherence, and prior rejection episodes (257, 258).

An immune-informed, individualized approach is particularly important in pediatrics because immune maturity, exposure history, and growth-related pharmacokinetics vary widely (259). Future directions include better noninvasive immune monitoring, harmonized definitions of rejection phenotypes, and phenotype-aligned interventions that reduce the long-term burden of immunosuppression while protecting the graft.

## 5 Conclusions and future directions

PHF is a complex, heterogeneous syndrome distinct from adult heart failure in etiology, myocardial biology, and clinical trajectory. Integrating cardio-immunology—the interplay between immune programs and neurohormonal pathways—adds a second, actionable axis for precision care, especially in myocarditis, MIS-C, postoperative inflammation, and infection-associated decompensation. Despite advances across multiple domains such as biomarkers, imaging, pharmacotherapy, device support, and transplantation, critical gaps persist: pediatric-specific biomarker reference ranges are lacking, inflammatory-immune phenotypes remain prospectively unvalidated, and treatment decisions rely heavily on extrapolated adult data. These limitations perpetuate practice heterogeneity and risk inappropriate immunomodulation.

Central to this framework is the recognition that immune contexts are transient, dynamic states that can superimpose upon underlying etiologies, requiring serial reassessment and context-appropriate therapeutic responses rather than fixed treatment algorithms based on etiology alone.

Addressing these challenges requires a coordinated three-phase research roadmap:

- Phase I: standardization. Establish international, age-specific reference ranges and harmonized sampling protocols for core inflammatory biomarkers (CRP, NT-proBNP, troponin, sST2, galectin-3).
- Phase II: validation. Prospectively validate inflammatory-immune phenotypes via multicenter cohorts linking operational criteria (clinical triggers, biomarker trajectories, imaging) to meaningful outcomes, thereby defining therapeutic windows.
- Phase III: implementation. Advance toward precision medicine by integrating multi-omics (proteomics, transcriptomics, metabolomics) to distinguish active immune injury from chronic remodeling and guide individualized therapy, alongside developing pediatric-specific immunomodulatory agents.

This roadmap provides a strategic pathway to transform PHF care from extrapolation-based practice into a phenotype-driven, evidence-based discipline aligned with contemporary pediatric heart failure consensus statements.
